# Extravasation of [^177^Lu]Lu-DOTATOC: case report and discussion

**DOI:** 10.1186/s13550-020-00658-6

**Published:** 2020-06-23

**Authors:** Anne Kirstine Arveschoug, Anne Charlotte Bekker, Peter Iversen, Henrik Bluhme, Gerda Elisabeth Villadsen, Peter Frøhlich Staanum

**Affiliations:** 1 Department of Nuclear Medicine and PET-Centre, Aarhus University Hospital, Palle Juul-Jensens Boulevard 165, 8200 Aarhus N, Denmark; 2 Department of Hepatology and Gastroenterology, Aarhus University Hospital, Palle Juul-Jensens Boulevard 99, 8200 Aarhus N, Denmark

**Keywords:** Extravasation, Peptide-receptor radionuclide therapy (PRRT), Kidney dosimetry, [^177^Lu]Lu-DOTATOC

## Abstract

**Background:**

In the case of extravasation of radioactive drugs used in peptide-receptor radionuclide therapy of neuroendocrine tumors, or in radionuclide therapy in general, rapid action is important to reduce or avoid complications. The literature on extravasation of drugs for radionuclide therapy is sparse. Based on the present case, we discuss handling and consequences of extravasation. Further, we demonstrate that dosimetry can aid in judging if the treatment of neuroendocrine tumors is satisfactory even after extravasation.

**Case presentation:**

A case of extravasation of [^177^Lu]Lu-DOTATOC with a treatment strategy involving exercise and elevation of the affected arm and application of a compression bandage and heating is reported. Redistribution of the drug is verified and quantified by whole-body imaging and quantitative SPECT/CT and measurements of the dose rate at contact with the injection site. [^177^Lu]Lu-DOTATOC was redistributed to tumors and organs within 1 day. The patient did not report any discomfort during or after hospitalization, and no side effects related to extravasation were observed. Quantitative SPECT/CT scans at the subsequent treatment cycle of the same patient were analyzed for a comparison between the treatments. Dosimetry showed the treatments were similar with respect to the kidney and tumor absorbed doses. The radiation dose to the epidermal basal layer near the injection site was estimated and found to be consistent with the lack of side effects.

**Conclusions:**

The treatment of extravasation was successful, and the redistribution of the drug can be easily verified through measurement of the dose rate at contact with the skin. From the results of dosimetry, it was assessed that no change of the treatment course was necessary to compensate for a possibly incomplete treatment as a result of the extravasation.

## Background

The use of peptide-receptor radionuclide therapy (PRRT) of neuroendocrine tumors (NETs) is increasing across the world, not at least since the NETTER trial demonstrated an increased survival when compared to best supportive care including octreotide long-acting repeatable (LAR) [1].

Extravasations of drugs for PRRT or radionuclide therapy in general are rare events. The literature on the subject is limited, yet valuable, as practical experiences and advice are important in order to take rapid action as soon as an extravasation is realized. In a publication from 2017 by van der Pol and colleagues [2], the consequences of radiopharmaceutical extravasation and possible therapeutic interventions were reviewed with mention of 10 cases of extravasation of therapeutic radiopharmaceuticals—in most cases with the isotope ^90^Y. More recently cases with ^177^Lu [3, 4], ^223^Ra [5], and an additional case with ^90^Y [6] have been reported. In some cases, little or no medical consequences of extravasation are reported, while in other cases severe skin damage can be observed. To the best of our knowledge, only one other case with extravasation of ^177^Lu-labelled peptides has been published [3], in spite of the fact that hundreds of daily treatment cycles with [^177^Lu]Lu-DOTATATE and [^177^Lu]Lu-DOTATOC take place in the PRRT setting in NET-centers all over the world today.

In the present work, we describe a case of extravasation of [^177^Lu]Lu-DOTATOC, which was realized shortly after injection. Treatment of the extravasation is described, and documentation of the redistribution as measured by dose rate measurements and quantitative SPECT/CT scans is reported and discussed in relation to the current literature.

## Case presentation

A 68-year-old female patient with a progressive midgut neuroendocrine tumor (NET) with liver and intraperitoneal dissemination was scheduled for peptide receptor radionuclide therapy (PRRT) with [^177^Lu]Lu-DOTATOC. Four cycles were planned with standard activity of 7.4 GBq [^177^Lu]Lu-DOTATOC and kidney protection during PPRT with an arginine/lysine mixture according to the EANM guidelines [7].

[^177^Lu]Lu-DOTATOC was administered through a peripheral venous catheter placed in the left cubital fossa in the first treatment cycle. After the injection of [^177^Lu]Lu-DOTATOC (7.5 GBq in 30 ml saline injected over 5 min) and a subsequent injection of 100 ml saline for rinsing of the syringe and the connecting hose, a swelling of the upper left arm was noted, and the patient confirmed a feeling of tenderness in the upper left arm. Extravasation was suspected and confirmed by whole-body scintigraphy as well as SPECT/CT of the left arm.

**Fig. 1:**
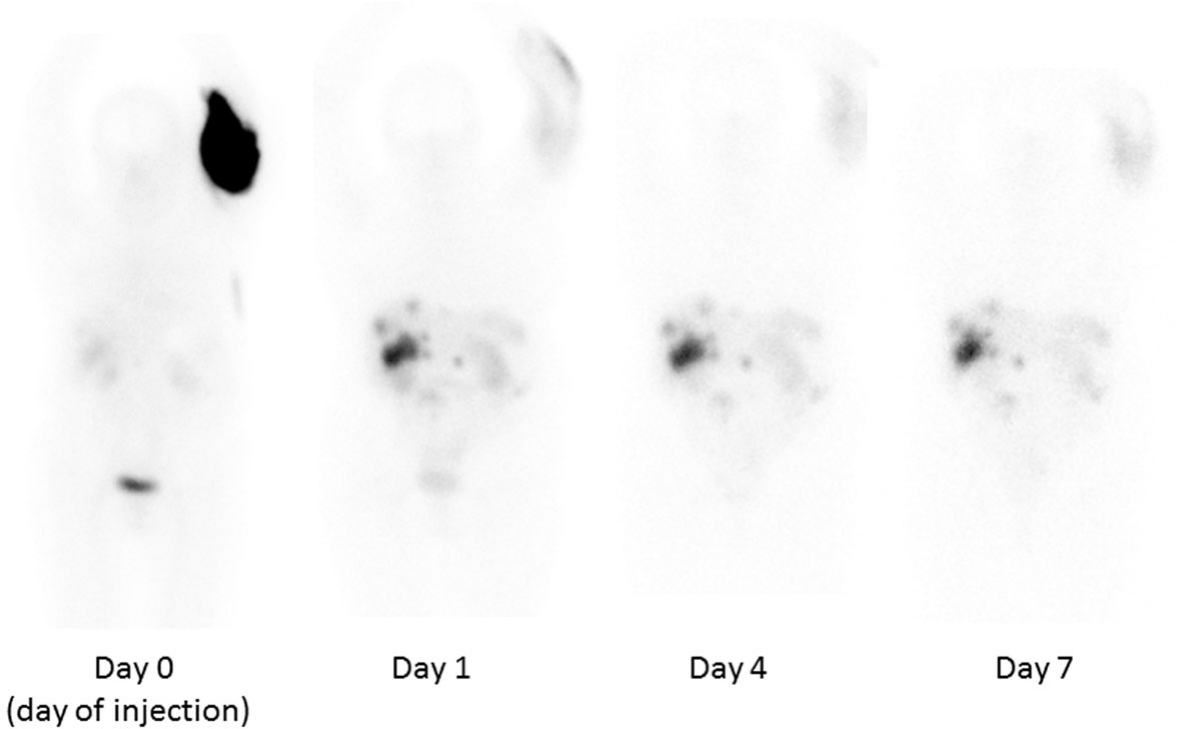
Anterior views of whole-body scintigraphies performed at day 0, day 1, day 4 and day 7. The activity initially located in the arm is redistributed to organs and tumors in the abdominal region

**Fig. 2:**
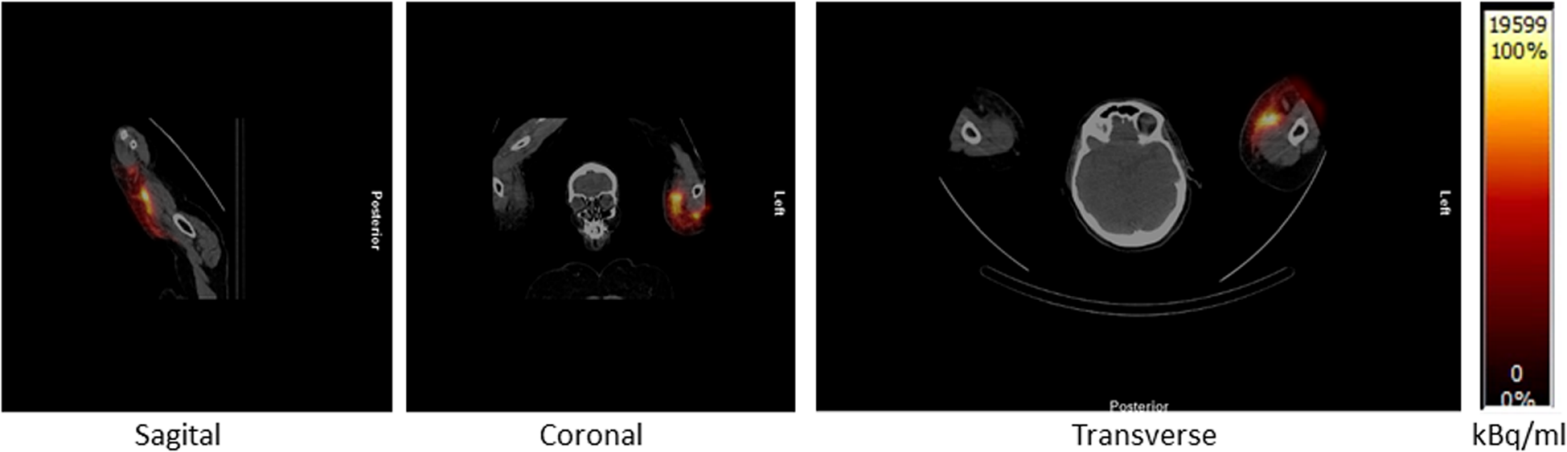
Distribution of treatment dose in the soft tissue of the arm at the day of treatment

The whole-body scintigraphy was initiated 83 min after injection start and showed a large concentration of activity in the upper left arm, while there was only little activity in the remainder of the body at this time point (Fig. 1). The SPECT/CT (started 110 min after injection start) showed activity in the subcutaneous tissue on both the medial and lateral side of the upper left arm (Fig. 2), and the later analysis of a quantitative SPECT reconstruction showed that more than half of the injected dose was located in the upper arm.

In order to stimulate the lymphatic drainage [2, 8], the patient was instructed to both elevate and exercise the affected arm by flexing the elbow, and a compression bandage with heated gel pads was applied to the relevant area. This stimulation was initiated shortly after confirmation of extravasation by the whole-body and SPECT/CT scans.

The standard protocol for nephroprotection with infusion of 25 g of lysine and 25 g of arginine dissolved in 1 l normal saline, and additionally 1 l of normal saline over 4 h was extended to 12 h with infusion of one additional solution of 25 g lysine and 25 g arginine in 1 l of saline. Finally, a separate infusion of 0.5 l saline with arginine and lysine over 2 h, 24 h after the treatment, was performed in order to extend the protection of the kidneys.

The day after the treatment (day 1), another whole-body scintigraphy and a quantitative SPECT/CT scan of the arm showed a dramatic decrease in total activity in the affected arm, and it was later estimated that less than 1% of the injected activity remained in the arm. The images showed high uptake in the metastasis in the liver and peritoneum correlating to the lesions of the pre-therapeutic [^68^Ga]Ga-DOTATOC PET/CT and a normal physiological uptake in the spleen, liver, and bladder.

Whole-body scintigraphies performed 4 and 7 days after injection showed a further decrease of the activity in the arm relative to activity in the abdominal region, see Fig. 1. SPECT/CT scans of the abdomen were also performed at day 4 and day 7 for dosimetry of kidneys and tumors as described below.

**Fig. 3:**
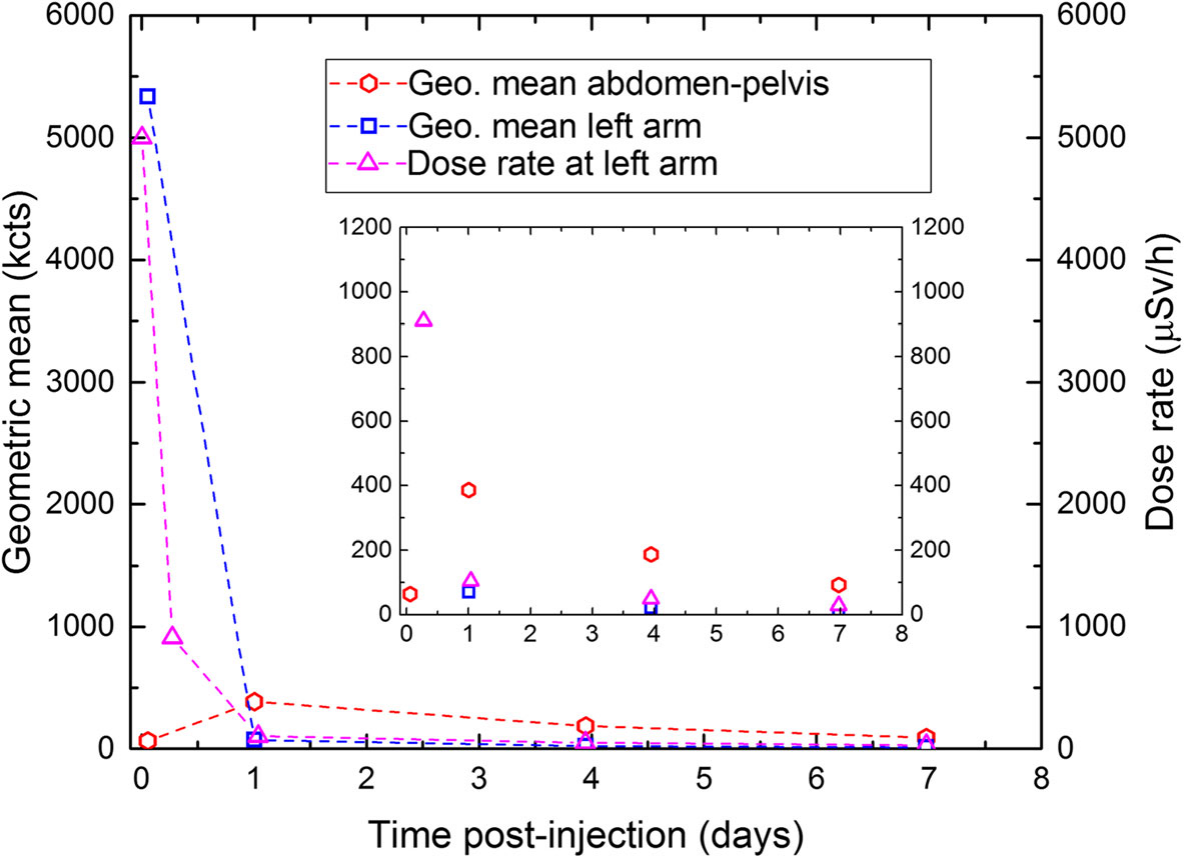
Geometric mean over the abdominal-pelvic region including liver, spleen, tumors and bladder (red hexagons; background corrected using a region-of-interest in the thorax) and over the left arm (blue squares; background corrected using a region-of-interest in the right arm) with units on the left axis. The dose rate with data from Table 1 is plotted as magenta triangles with units on the right axis (the scales on the left and right axis are the same by coincidence). The dashed lines are intended to guide the eye. The inset shows a zoom of the main graph

The remaining activity in the arm was also assessed at different time points by measurement of the dose rate close to the skin surface of the affected upper arm using a Rados RDS-100 survey meter (Table 1). The temporal development of activity in the left arm, the abdominal-pelvic region, and the dose rate at contact with the arm is shown in Fig. 3, which indeed demonstrates a rapid decline of activity in the arm. The temporal development of the geometric mean over the arm and the dose rate is in very good agreement. In addition, delayed uptake of activity in the abdominal organs and tumors and the bladder can be observed.

**Table 1:** Dose rate at contact with the injection site in the left arm

Time post-injection (hours)	Dose rate (μSv/h)
0.1	5000
6.6	910
24.9	104
94.7	49
167.4	29

The patient had no symptoms from the affected arm, or in general, and was discharged 2 days after the PRRT. The patient was contacted daily by the outpatient clinic in the week after the discharge, and the patient did not report any discomfort, which was also not the case during the 1-year follow-up. There were no complications in the following three treatment cycles, where PRRT was given in a central venous catheter.

### Dosimetry

The SPECT/CT scans were recorded using a Siemens Symbia T16 SPECT/CT scanner (Siemens Medical Solutions USA, Inc.; 208 keV photopeak, medium energy collimator, 40 s/view (day 0 and day 1) or 60 s/view (day 4 and day 7), 32 views per detector, 128 × 128 matrix, 4.8 mm pixel size and CT with 110 kV and 60 mAs (quality ref.)) and reconstructed as a quantitative SPECT/CT scan with prior calibration following Beauregard et al. [9]. For analysis of the radiation dose to the kidneys and selected tumors, the scans over the abdomen at days 1, 4, and 7 after injection were reconstructed with pixel values equal to activity concentration in units of 100 Bq/ml.

**Fig. 4:**
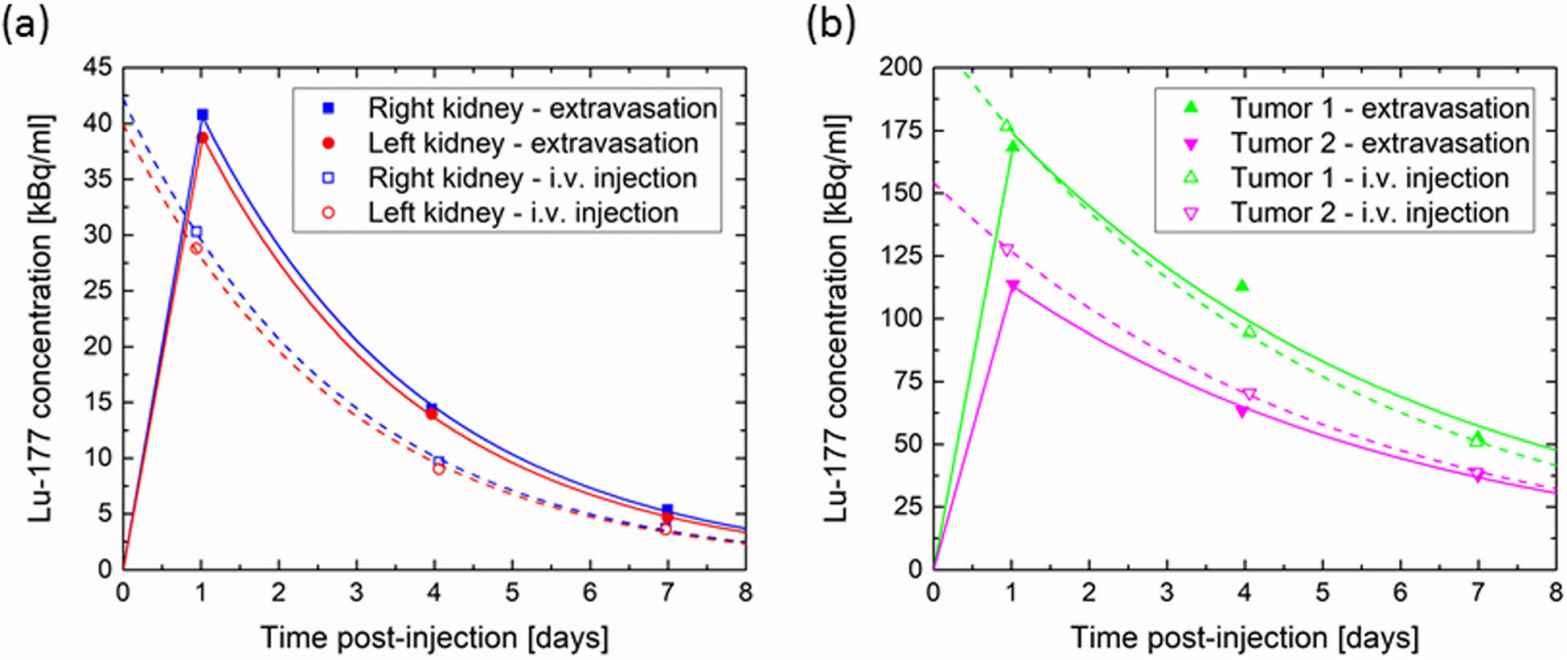
Mean activity concentration in (**a**) the left and right kidneys and (**b**) two tumors in the liver after the first and second PRRT cycle. The areas under the solid and dashed curves are proportional to the absorbed doses given in Table 2

The kidneys were delineated manually in Hermes Hybrid Viewer (Hermes Medical Solutions AB, Sweden) on each CT scan; the volume was transferred to the corresponding quantitative SPECT reconstruction, and the mean concentration of ^177^Lu in each kidney was determined. The mean concentration was converted to units of *kilobecquerel per milliliter* (kBq/ml) and plotted versus time post-injection (Fig. 4a). Normally, the data are fitted to a mono-exponential decay function, and the time-integrated activity concentration is determined as the area under this curve (AUC, area-under-curve). The absorbed dose is calculated by multiplying AUC with the three factors 1.95 mGy ml/(kBq d) (assuming total beta radiation absorption with mean beta energy 0.1479 MeV [10] in the kidney tissue with density 1.05 g/ml [11]), 1.05 (accounting for gamma radiation contributions [12]), and 1/0.85 (to account for partial volume effects of the delineated volume [13]). In the first treatment cycle of the present case, a mono-exponential decay fit would, however, lead to an overestimate of the dose as the uptake in the kidneys are delayed in comparison with an i.v. injection. Therefore, the initial uptake is approximated by a straight line from zero activity at injection to the first data point, and only after the first data point a mono-exponential decay function is applied (see Fig. 4a). The linear approximation results in a correction of −0.7 Gy for both kidneys as compared to a calculation where only a mono-exponential decay function is applied.

Two tumors in the liver with high uptake were delineated by setting a 50% threshold of the maximum concentration in each tumor as they were not distinguishable from normal liver tissue on the CT scan. The mean concentration within the thresholded volumes at days 1, 4, and 7 was plotted against time post-injection and the AUC determined, again using a linear approximation until the first data point and a mono-exponential decay function thereafter (see Fig. 4b). The AUC was multiplied by the same factors as for the kidneys to derive a measure of the absorbed dose of the two tumors. The dose derived in this way is not necessarily equal to the mean dose of the tumor; it is a dose measure which enables a comparison between the two treatment cycles. The absorbed dose, the specific dose, and the effective decay time of each kidney and tumor are given in Table 2.

**Table 2:** Dosimetry of the left and right kidneys and two tumors in the liver

		First treatment (extravasation)	Second treatment (i.v. injection)
	Activity (GBq)	7.5	7.6
Right kidney	Absorbed dose (Gy)	3.3	2.9
	Specific dose (Gy/GBq)	0.45	0.37
	Effective half-life (days)	2.0	1.9
Left kidney	Absorbed dose (Gy)	3.1	2.7
	Specific dose (Gy/GBq)	0.42	0.36
	Effective half-life (days)	2.0	2.0
Tumor 1	Absorbed dose (Gy)	25	25
	Specific dose (Gy/GBq)	3.3	3.3
	Effective half-life (days)	3.7	3.4
Tumor 2	Absorbed dose (Gy)	16	19
	Specific dose (Gy/GBq)	2.1	2.5
	Effective half-life (days)	3.7	3.5

Quantitative SPECT/CT scans at days 1, 4, and 7 after the second PRRT of the patient, 9 weeks after the initial treatment, were analyzed as above (without linear extrapolation to the first data point), and data are also shown in Fig. 4 and Table 2.

The doses to the kidneys and tumors are in very good agreement between the first and the second treatment taking into account the natural variation between treatments, the uncertainty of the dosimetry method, and in particular the uncertainty related to the linear approximation from injection to the first data point after the first treatment. This leads us to conclude that no change of the treatment course was necessary to compensate for a possibly incomplete treatment as a result of the extravasation. The third and the fourth treatments were carried out as planned. After these treatment cycles, only a single quantitative SPECT/CT scan was performed at day 1, and the same effective half-life as after the second treatment was assumed for kidney dosimetry. At the third treatment with 7.9 GBq [^177^Lu]Lu-DOTATOC, the absorbed doses of both kidneys were 3.1 Gy, and at the fourth treatment with 8.0 GBq [^177^Lu]Lu-DOTATOC, the absorbed doses of the right and left kidneys were 3.3 Gy and 3.1 Gy, respectively.

The absorbed dose to the skin epidermal basal layer near the injection site can be estimated as follows from the dose rate measurements and a quantitative SPECT/CT scan. By assuming that the temporal evolution of the activity near the injection site follows the dose rate measurements and scaling these data to the concentration found from a quantitative SPECT/CT scan of the arm, a time-activity curve can be generated and from this the AUC and the absorbed dose to the volume containing the extravasate be determined. The scaling factor is determined using the SPECT/CT scan at day 1, and the dose rate measurement at 24.9 h performed immediately before the scan. At this time point, the mean concentration in a volume defined by a 50% threshold of the local maximum was 163 kBq/ml (the volume is 14 ml), and hence, the scaling factor is 1.567 kBq h/(ml μSv). The AUC is calculated using the trapezoidal rule between the data points and extrapolating to time zero and infinity by using the slope found from linear interpolation between the first two and the last two data points, respectively. By assuming total absorption of beta-radiation in tissue of density 1.0 g/ml, the AUC should be multiplied by 85.3 μGy ml/(kBq h) to calculate the absorbed dose, which is then found to be 6 Gy. The dose to the epidermal basal layer is approximately half of this dose, i.e., 3 Gy, as the epidermal basal layer is only irradiated from one side [14].

## Discussion

As already mentioned, only one of the cases of extravasation reported in the literature is concerned with a ^177^Lu-labelled peptide. Tylski and colleagues [3] described a case with [^177^Lu]Lu-DOTATATE, where they found a quick elimination of the drug from the arm with an effective half-life of 3 h and that the elimination could be increased by local warming and repeated massage of the injection site. This patient was also without any clinical signs of radiation damage.

A similar case with [^90^Y]Y-DOTATOC was reported by Terwinghe et al*.* [8]. They also found a remarkable decrease in activity in the arm, which at first contained all of the injected 3.5 GBq [^90^Y]Y-DOTATOC. The dose rate at contact with the arm fell from 75 to 1 mSv/h in a day, and Bremsstrahlung images showed significantly lower retention of [^90^Y]Y-DOTATOC the day after treatment, with a decrease of 91% from the day of treatment. Terwinghe et al. concluded at that time, in 2012, that the relatively small molecule [^90^Y]Y-DOTATOC (molar mass 1.5 kDa) has a lower retention in subcutaneous tissue than heavier molecules like the monoclonal antibody [^90^Y]Y-Ibritumomab tiuxetan (Zevalin, used for treatment of non-Hodgkin’s lymphoma, molar mass about 148 kDa) as there had been reports of pronounced local radiation damage and skin necrosis after extravasation with [^90^Y]Y-Ibritumomab tiuxetan [15, 16].

In contrast to the conclusion of Terwinghe et al., a case of extravasation with the relatively large [^177^Lu]Lu-PSMA molecule (molar mass about 84 kDa) was reported by Schlenkhoff and colleagues in 2017 [4], where no symptoms from the arm and no complications afterwards were observed. The case was very similar to the present one, but they took a different approach to treatment with attempts to squeeze the fluid back via the catheter, followed by heating of the injection area for 12 h, and finally by cooling the area and no movement of the affected arm. They also found a rapid decline of activity in the arm and a normal distribution of [^177^Lu]Lu-PSMA after 20 h. Furthermore, the reports of skin burns [14] and development of subcutaneous squamous carcinoma [5] after extravasation with the small molecules [^131^I]I-metaiodbenzylguanidine (MIBG, 0.3 kDa) and ^223^RaCl_2_ (Xofigo, 0.3 kDa), respectively, are also in contrast to the conclusion of Terwinghe et al.

The lack of complications after extravasation of [^177^Lu]Lu-PSMA and the serious complications observed after extravasation of [^131^I]I-MIBG and ^223^RaCl_2_ are inconsistent with the hypothesis that extravasation of small molecules should result in little radiation damage of the skin due to a lower retention in subcutaneous tissue. Instead, we note that in the above-mentioned cases [3, 4, 8], as well as the present case, where no complications were observed, there was an immediate treatment by local heating, massage, or exercise, as recommended both in the EANM procedure guidelines for treatment with [^90^Y]Y-Ibritumomab tiuxetan (Zevalin®) [17] and ^223^RaCl_2_ (Xofigo®) [18] and in the product characteristics of Lutathera® ([^177^Lu]Lu-DOTATATE) [19]. In most of the reported cases with serious radiation damage [5, 14, 15, 16], no immediate treatment was initiated, while in one recent case with [^90^Y]Y-Ibritumomab tiuxetan [6], a patient was in need for surgery with removal of necrotic skin and soft tissue even though extravasation was quickly realized and early treatment was attempted by aspiration of the puncture site and massage of the arm. These observations from cases with and without radiation damage indicate that immediate and efficient treatment is decisive for the outcome after extravasation regardless of the size of the molecule.

It is also noteworthy that in the cases without tissue damage [3, 4, 8], relatively large volumes of saline was co-infused with the therapeutic drug leading to a somewhat lower radioactivity concentration than in cases with tissue damage. In particular, the infusion of 1.2 GBq of ^90^Y in 10 ml in Zevalin treatments results in a relatively high concentration of the high-energy beta-emitter ^90^Y and hence a relatively high absorbed dose near the injection site in the arm.

In the present case, we found that after immediate initiation of massage, heating, and exercise of the arm, the radio-therapeutic drug was redistributed to the organs and tumors as we would expect after a successful i.v. injection. The successful treatment of the extravasation could easily be verified as a drop in dose rate measured by using a survey meter. The absorbed dose to the epidermal basal layer was estimated to 3 Gy. At this dose, no damage to the skin is anticipated according to [3], where a dose of 2.8–7.8 Gy was estimated and no clinical signs of irradiation were found, and according to Fig. 1 in [6] where the mildest clinical response shown, a transient depilation, occurs at a dose of 4–6 Gy. In other cases where higher absorbed doses of 10–20 Gy (acute) plus 12–16 Gy (low dose rate irradiation) [14], 20–40 Gy [16] and 43 Gy [6] have been estimated, a moist or wet desquamation, or even skin necrosis was observed.

Further, in the present case, dosimetry of the kidneys and tumors after the first and the second PRRT cycle documented that even after extravasation the absorbed doses to the tumors were similar to those after a successful injection, and therefore, no additional PRRT cycle was scheduled for the patient after the extravasation.

As a growing amount of therapeutic injections in nuclear medicine are being performed, there is an increasing need for guidelines regarding extravasation both with respect to prevention and to early and delayed treatment. As a consequence of the present case, we have adapted some of the advices mentioned by Williams and coworkers [16] in an institutional guideline for our different radionuclide therapies. In order to prevent further incidents, we have clarified our procedures for intravenous therapy so that the needle is now being placed in the upper third of the lower arm, avoiding the joint area, and ultrasound is being used in difficult cases. In patients with a known history of difficult intravenous access, a central venous catheter is used for the therapy. The use of a new venipuncture site in large veins, including central venous catheter, and checking for blood return was already incorporated in our procedures. Regarding the early treatment of extravasation, we recommend massage, heating, exercise and elevation, and monitoring by a survey meter as mentioned above. With appropriate measurements, it is feasible to estimate radiation dose to the epidermal basal layer and hence the severity of the extravasation and ultimately the prognosis.

## Data Availability

The datasets used or analyzed during the current study are available from the corresponding author on reasonable request.

## Abbreviations

AUC: Area-under-curve
CT: Computed tomography
DOTATATE: (Dota^0^-Tyr^3^)octreotate
DOTATOC: (Dota^0^-Phe^1^-Tyr^3^)octreotide
LAR: Octreotide long-acting repeatable
MIBG: Metaiodobenzylguanidine
NET: Neuroendocrine tumor
PRRT: Peptide-receptor radionuclide therapy
PSMA: Prostate-specific membrane antigen
SPECT: Single-photon emission computed tomography
